# Symptom clusters and resting cardiovascular autonomic measures in adolescents: From acute concussion to recovery

**DOI:** 10.14814/phy2.70114

**Published:** 2024-11-03

**Authors:** Wenjie Ji, Haley M. Chizuk, John J. Leddy, Sue A. Sisto, Mohammad N. Haider

**Affiliations:** ^1^ Department of Rehabilitation Science School of Public Health and Health Professions, State University of New York at Buffalo Buffalo New York USA; ^2^ UBMD Orthopaedics and Sports Medicine, Jacobs School of Medicine and Biomedical Sciences State University of New York at Buffalo Buffalo New York USA

**Keywords:** autonomic nervous system, brain concussion, post concussion symptom

## Abstract

Sport‐related concussion (SRC) is associated with cardiovascular autonomic nervous system (ANS) dysfunction. This study examines resting cardiovascular ANS activity in adolescents with SRC compared to controls early post‐injury and after clinical recovery, analyzing its correlation with symptom severity and recovery outcomes. Cardiovascular ANS function was evaluated using heart rate variability (HRV), systolic blood pressure variability (SBPV) and baroreflex sensitivity (BRS). Symptoms were assessed via the Post‐Concussion Symptom Scale, and recovery outcomes were categorized by recovery duration. Following acute SRC, no significant differences in HRV, SBPV or BRS were found between SRC and control groups, nor between those with delayed or normal recovery. Post‐recovery, SRC participants had higher low frequency (LF) SBPV than controls and their initial assessment. When concussed participants were symptomatic, LF SBPV correlated directly with overall, cognitive, and fatigue symptom severity, while high frequency (HF) HRV inversely correlated with affective symptoms (Spearman's rho: 0.4–0.6). Resting cardiovascular ANS function remains unchanged in adolescent athletes acutely after SRC, suggesting it has limited diagnostic and prognostic potential. Although some correlations between individual symptom domains and ANS activity were observed, they were not significantly different from asymptomatic controls, limiting the ability to interpret these findings.

## 1 INTRODUCTION

Sport‐related concussion (SRC) is a mild traumatic brain injury (mTBI) caused by a direct blow to the head, face, neck or the body during sport or recreational activities (McCrory et al., 2017). Autonomic nervous system (ANS) function is typically assessed via non‐invasive cardiovascular measurements (Chong & Schwedt, 2018; Darling et al., 2014; McCrea et al., 2017; Polak et al., 2015), and SRC have been found to cause dysregulation of both the sympathetic (SNS) (Johnson et al., 2020) and parasympathetic (PNS) (Johnson et al., 2018) branches. This dysregulation has been found to persist beyond clinical recovery (Kamins et al., 2017; Memmini et al., 2021). ANS dysregulation is also thought to impair cardiovascular function, impeding exercise performance in concussed athletes (Heyer et al., 2016; Hinds et al., 2016). Previous studies have suggested an association between cardiovascular ANS tone and clinical characteristics of concussion (McCrory et al., 2017), but these findings are conflicting (Coffman et al., 2021; Memmini et al., 2021; Paniccia et al., 2018; Purkayastha et al., 2019). While some research has found that a decrease in PNS tone is associated with more symptoms (Coffman et al., 2021), other studies have reported that a decrease in PNS tone is associated with fewer symptoms (Paniccia et al., 2018). Additionally, in adults with non‐SRC, increased PNS activity has been positively correlated with greater levels of anxiety and depression (Mercier et al., 2022). However, in the general population, higher‐level evidence indicates that emotional disturbances are associated with reduced PNS activity and increased SNS activity (Thayer & Lane, 2009).

Some non‐invasive measures of cardiovascular ANS tone include heart rate variability (HRV), blood pressure variability (BPV) and baroreflex sensitivity (BRS). HRV is defined as the fluctuations between consecutive R–R intervals (Electrophysiology TFotESoCtNASoP, 1996), and broad evidence supports that the root mean square of successive differences (RMSSD) and the high frequency (HF: 0.15–0.4 Hz) component of HRV (HF HRV) reflect changes in cardiovascular PNS activity (Cowan, 1995; Joyner, 2016; Malik & Camm, 1993; Stauss, 2003). Similarly, BPV is the fluctuation between blood pressure (BP) waveforms (Gulli et al., 2003), and the low frequency (LF: 0.05–0.15 Hz) component of systolic BPV (LF SBPV) is a useful biomarker for assessing cardiovascular SNS activity (Diedrich et al., 2003; Ellingson et al., 2022; Parati et al., 2018). Although there is conflicting evidence, the LF component of HRV (LF HRV) is generally believed to reflect both SNS and PNS activity (Billman, 2013; Houle & Billman, 1999). BRS provides extra information on ANS‐mediated cardiovascular regulation (Hilz & Dutsch, 2006; Parati et al., 2000). Baroreceptors in the carotid sinuses and aortic arch detect changes in BP and provide negative feedback to restore it to baseline levels (La Rovere et al., 2008). However, how SRC affects BRS has not been extensively studied, and the existing results are conflicting (LA Fountaine et al., 2019; Mercier et al., 2022; Strachan, 2013). Several invasive techniques have also been used to assess cardiovascular ANS tone, such as microneurography, but they are not a focus of this manuscript due to their decreased potential of use in an outpatient clinic setting (Carter, 2019; Metelka, 2014).

The clinical presentation of acute SRC is diverse, with athletes reporting a combination of physical (somatic), cognitive, fatigue and mood‐related symptoms (Langdon et al., 2020). Recovery from SRC also varies, with some athletes recovering within a few weeks, while others experience Persisting Post‐Concussion Symptoms (PPCS) (Patricios et al., 2023). A comprehensive understanding of how the cardiovascular ANS interacts with individual clinical characteristics and outcomes may help explain the complexity of autonomic dysfunction after SRC. The aim of the current study was to compare cardiovascular ANS tone in adolescents with SRC versus matched controls early after injury and again after clinical recovery, and to assess the association with clinical variables. We hypothesized that resting cardiovascular ANS measures would differ early after injury but not after clinical recovery, and that ANS tone during the acute injury phase would have significant associations with symptom reporting and recovery outcomes.

## 2 MATERIALS AND METHODS

This analysis is part of a larger prospective case control study approved by the University at Buffalo's Institutional Review Board (Approval Number: MOD00007947). Adolescents with SRC were recruited from three university‐affiliated sports medicine clinics in Buffalo, NY. At their first clinical visit, a research assistant explained the study, screened for eligibility, and obtained written consent in a HIPAA‐compliant setting. Parental consent and participant assent were obtained for all minors (aged 13–17). The first research visit (V1) occurred within 10 days of injury. Injuries were diagnosed and managed by a sports medicine physicians based on international guidelines (Patricios et al., 2023). Participants followed up with their physician weekly according to standardized, recommended clinical protocols until they were cleared to begin a return‐to‐play protocol (McCrory et al., 2017; Patricios et al., 2023). Clinical recovery was defined as the resolution of concussion‐like symptoms to baseline, a physical examination within normal limits, and demonstration of normal exercise tolerance on the Buffalo Concussion Treadmill Test (BCTT) (Haider et al., 2017; Kumar et al., 2023). Participants returned for their post‐recovery visit (V2) within 1 month of successfully completing their return‐to‐play protocol. Sex‐, age‐, and competition level‐matched healthy controls were recruited from local high schools and sports teams. Healthy controls were asked to return for V2 approximately 6–8 weeks after V1 to reflect a standard 1‐month SRC recovery time and 2‐week return‐to‐play protocol.

### 2.1 Participants

The inclusion criteria for the concussion group were: (1) obtained a concussion during a sport‐related activity; (2) aged 13–18 years; and (3) sustained injury within 10 days prior to V1. Participants were excluded if they had any one of the following: (1) more severe injury than mTBI corresponding to a Glasgow Coma Scale <13, loss of consciousness for >30 min or post‐traumatic amnesia for >24 h, or a focal neurological deficit consistent with an intracerebral lesion (e.g., unilateral weakness, dilated pupil); (2) active substance abuse/dependence; (3) history of moderate or severe TBI; (4) more than 3 previous concussions; and (5) currently on a beta‐ or calcium‐blocker or medications that affect ANS function (e.g. ADHD stimulants and mood‐stabilizers). For the control group, the inclusion criteria were: (1) aged 13–18 years; (2) no history of concussion within the past year; and (3) played at least one organized sport (to control for athletic status). Exclusion criteria for controls were identical to participants with SRC.

### 2.2 Symptom classification

The Post‐Concussion Symptom Scale (PCSS) was used to report concussion‐like symptoms that were categorized into 4 domains: physical, cognitive, affective, and fatigue (Table 1) based on the symptom profiles of a prior version of the PCSS that only had 19 symptoms (Merritt et al., 2015). The PCSS is a self‐report measure that was developed to assess SRC‐related symptoms (Karr et al., 2023). The version we used comprises 22 items, which are evaluated on a 7‐point scale ranging from 0 to 6, with 0 indicating no symptoms and 6 indicating severe symptoms, and the measure has excellent internal consistency (Langevin et al., 2022).

**TABLE 1:** PCSS symptom domains and their associated items.

Physical (10 items)	Cognitive (6 items)	Affective (4 items)	Fatigue (2 items)
Headache Pressure in head Neck pain Nausea or vomiting Dizziness Blurred vision Balance Sensitivity to light Sensitivity to noise Trouble falling asleep	Feel slowed down Feel like “in a fog” “Do not feel right” Difficulty concentrating Difficulty remembering Confusion	More emotional Irritability Sadness Nervous or anxious	Fatigue or low energy Drowsiness

### 2.3 Experimental procedure

Concussed and control participants were asked to complete two visits with identical experimental procedures. Participants were instructed to refrain from alcohol, caffeine, and exercise for 12 h and food for 2 h prior to their research visits. Room temperature was controlled and ranged from 20° to 23°C and humidity was controlled between 15% and 25%. Participants assumed a supine position and were instrumented with an ECG, photoplethysmograph (PPG) and breathing mask. ECG and PPG readings were visually inspected for irregularities before recording to ensure recognition of any potential abnormalities. Breathing rates were not controlled. Cardiovascular parameters were recorded for a total of 10 min but only 5 min (Minute 4–9) were used for analysis (Gall et al., 2004). The final minute analysis was excluded because participants were informed that they had 30 s remaining, which may have affected their cardiovascular, respiratory and ANS activities.

### 2.4 Instrumentation and measurements

#### 2.4.1 Data acquisition

BIOPAC Systems, Inc. MP160 system was used for data acquisition and analysis of physiological data. The MP160 is a 16‐channel core system with a high‐level transducer interface module that uses AcqKnowledge 5.0 software for visualization and processing.

#### 2.4.2 ECG

Bionomadix 3‐lead ECG (DA100C, Biopac Systems, Goleta, CA) was used to obtain the R–R interval. The sampling rate was set at 1000 Hz.

#### 2.4.3 HRV analysis

Kubios HRV Software 5.0 with built‐in tools was used for ECG clean‐up, including a QRS detector, beat‐to‐beat analysis, and autocorrection for ectopic beats. Time‐ (RMSSD) and frequency‐ (HF; 0.15–0.4 Hz) domain measures were calculated from the spectral analysis of the R–R interval through Kubios HRV software (Tarvainen et al., 2002). Normalized HF HRV (HF HRV n. u.) was calculated by dividing raw HF by (raw HF + raw LF).

#### 2.4.4 PPG

ClearSight photo‐plethysmograph by Edwards Lifescience Inc., Irvine, CA (formerly Nexfin BMEYE, Amsterdam, Netherlands) was used on the left index finger (2nd metacarpal region) to obtain blood pressure waveforms. As a quality control measure, calibration using the Physiocal™ vascular unloading algorithm was used prior to each physiological test. The sampling rate was set at 250 Hz.

#### 2.4.5 Respiration

Participants breathed through a one‐way valve (Hans Rudolph, Kansas City, KC) with a nose clip. Respiratory rate (RR), tidal volume and minute ventilation were obtained using a pneumotach (Pneumotach Transducer, BIOPAC Systems Inc., Goleta, CA). The sampling rate was set at 62.5 Hz.

#### 2.4.6 SBPV analysis

SBPV was processed using the methods previously reported in MATLAB (Lucci et al., 2021). Occasional ectopic beats were removed by substituting them with average values of the resting condition, and significant trends were eliminated by subtracting the best‐fit polynomial function. The LF band was identified as 0.05–0.15 Hz using Fast Fourier Transform (Lucci et al., 2021). Subsequently, the absolute power of the LF SBPV was calculated using power spectral density.

#### 2.4.7 BRS analysis

BRS was processed using transfer function analysis via MATLAB (La Rovere et al., 2008). BRS is represented by the average gain between changes in systolic BP and the R–R interval within the frequency range of 0.07–0.14 Hz, a range that is reliable for BRS assessment as it is not significantly influenced by RR (La Rovere et al., 2008). Both the raw ECG and beat‐to‐beat BP data were down sampled to 4 Hz to align the two datasets for this analysis (Ondrusova et al., 2017).

### 2.5 Statistical analyses

No a priori sample size estimation was performed and this study utilized a convenience sample of adolescents who were participating in a larger study looking at exercise intolerance. The normality of continuous variables was assessed using a Shapiro Wilk test. Independent samples *t*‐tests and paired t‐test were used to compare group and within subject differences when the data were normally distributed. Mann Whitney U tests and Wilcoxon signed rank test were used when the data were not normally distributed. *χ* ^2^‐tests were used to compare categorical variables. Demographics and clinical characteristics were compared between groups. Cardiovascular parameters were compared within subjects across the two visits and between groups at both visits while controlling for RR using mixed effects linear regression. Only participants with SRC were used when measuring association between cardiovascular ANS parameters and symptom subtypes since all other subgroups reported minimal symptoms (i.e. almost everyone reported 0 on the PCSS). Pearson's correlation was used to analyze the relationship between symptoms and RMSSD, while Spearman's correlation was employed to examine associations with HF HRV, LF SBPV and BRS. The strength of the correlations was interpreted by referring to previously published statistical tutorials (Schober et al., 2018). Participants with SRC were then stratified based on the occurrence of PPCS (symptoms <28 days versus ≥28 days). Cardiovascular ANS variables were compared between participants with PPCS and without. A *p*‐value <0.05 was considered statistically significant for most analyses except the linear regressions with two variables. In these cases, a Bonferroni correction was applied and a p‐value of <0.025 was considered significant. All analyses were performed using SPSS Version 29 (IBM Corp, Armonk, NY).

## 3 RESULTS

**FIGURE 1:**
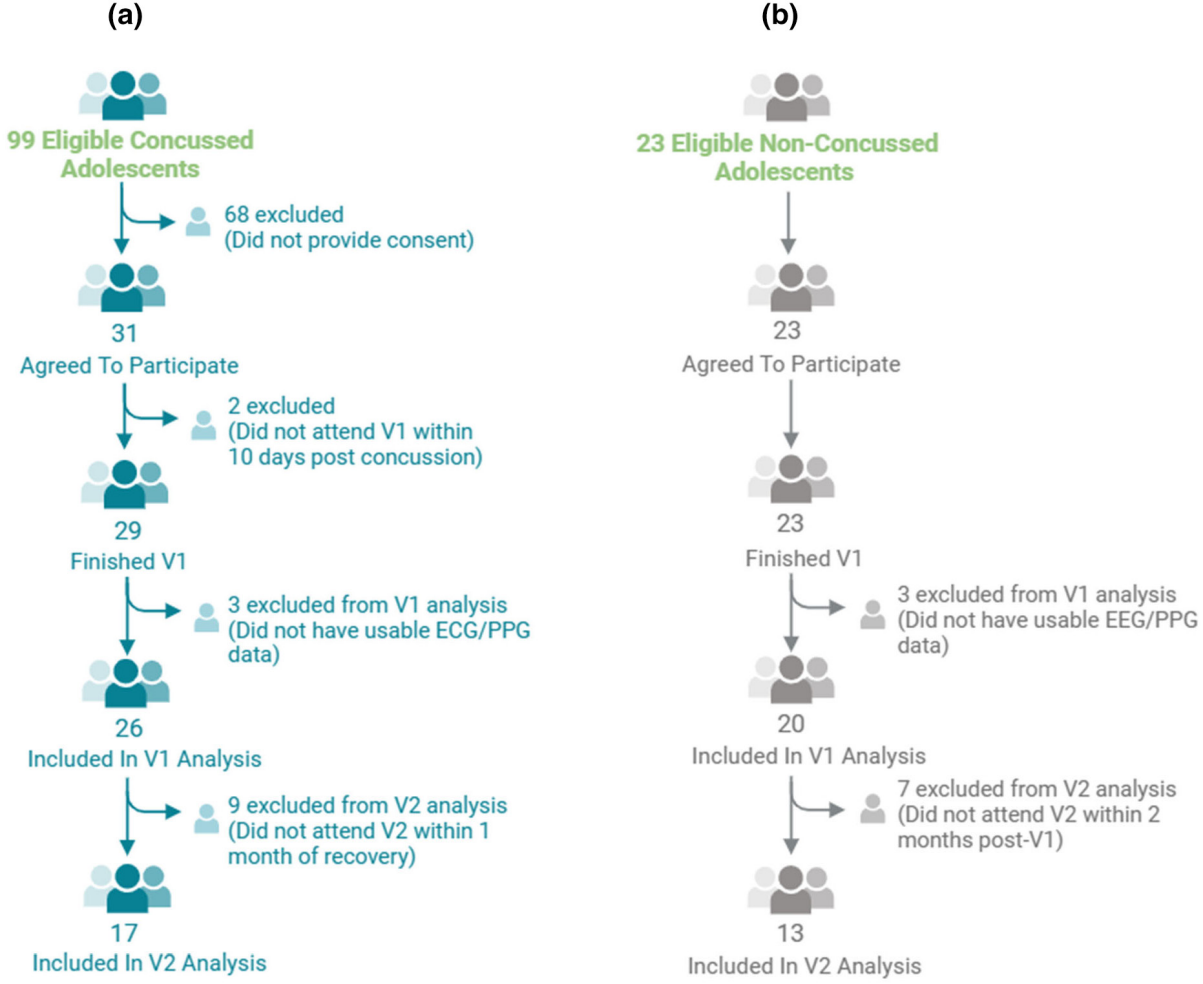
Framework of participants inclusion. (a) Framework of the inclusion of sport concussion group; (b) Framework of inclusion of healthy control group; ECG, electrocardiogram; HCG, healthy control group; PPG, photoplethysmography; SCG, sport concussion group; V1, visit 1; V2, visit 2.

Out of 99 eligible adolescents seen at the clinics from September 2016 to March 2020, 31 provided consent. Two participants that provided consent were unable to attend V1 within 10 days of injury and were excluded. Out of the 29 adolescents who performed the research study, 3 did not have usable ECG/PPG measurements (>5% noise on waveforms) and were excluded. Hence, 26 adolescents with SRC comprised the Sport Concussion Group (SCG) at V1. One participant in the SCG did not complete the PCSS at V1 and was not included in the subsequent symptom domain comparison. Twenty‐three healthy adolescents provided consent and performed the research procedures, however, 3 did not have usable ECG/PPG recordings and were not included in the analysis. Hence, 20 participants comprised the Healthy Control Group (HCG) at V1. Nine participants in the SCG did not schedule a research visit within 1 month of return‐to‐play. Additionally, 7 participants in the HCG were unable to schedule a research visit between 6 and 8 weeks since V1. Figure 1 illustrates the framework of participants' inclusion. No difference in age or sex was observed between participants who were included or excluded from the analyses.

Group‐wise demographics are provided in Table 2. Groups did not differ in age, sex, height, or weight, except that the SCG reported having more previous concussions and reported more concussion symptoms. The HCG also had a longer duration between V1 and V2 compared to the SCG, but this did not reach statistical significance.

**TABLE 2:** Participant demographics and clinical characteristics.

	SCG *n* = 26	HCG *n* = 20	*p*‐value
Age in years, mean (SD)	15.3 (1.2)	15.9 (1.4)	0.152
Sex, n (%) male	15 males (57.5%)	12 males (60%)	0.875
Height in meters, mean (SD)	1.72 (0.14)	1.68 (0.12)	0.432
Weight in kilogram, mean (SD)	69.37 (13.09)	67.22 (12.89)	0.563
Days between V1 and V2, mean (SD)	31 (17)	41 (8)	0.055
Days between injury and V1, mean (SD)	7 (2)	‐	‐
Previous Concussion, n (%)			**0.003**
0	8 (30.8%)	17 (85.0%)	
1	13 (50.0%)	1 (5.0%)	
2	3 (11.5%)	1 (5.0%)	
3	2 (7.7%)	1 (5.0%)	
Total symptom severity, mean (SD)	24.8 (3.1)	0.8 (3.0)	**< 0.001**
Physical symptom severity, mean (SD)	10.5 (1.2)	0.3 (1.1)	**< 0.001**
Cognitive symptom severity, mean (SD)	8.7 (1.3)	0.2 (0.9)	**< 0.001**
Affective symptom severity, mean (SD)	2.5 (0.7)	0.1 (0.3)	**< 0.001**
Fatigue symptom severity, mean (SD)	3.1 (0.6)	0.2 (0.9)	**< 0.001**

*Note*: Bolded values indicate a significant finding.

Abbreviation: HCG: healthy control group; SCG: sport concussion group; SD: standard deviation; V1: visit 1; V2: visit 2.

Cardiovascular data at V1 and V2 are presented in Table 3. No significant differences were seen at either timepoint except that the SCG had higher LF SBPV at V2 (after recovery) when compared to the HCG. Regarding within‐subjects change of ANS measures from V1 to V2, no differences were observed in HCG between timepoints in HF HRV (*p* = 0.972), RMSSD (*p* = 0.507), LF SBPV (*p* = 0.753) and BRS (*p* = 0.200). No differences were observed in SCG in HF HRV (*p* = 0.093), RMSSD (*p* = 0.619) or BRS (*p* = 0.357) but there was a difference in LF SBPV with SCG showing a significant increase in resting LF SBPV after recovery (*p* = 0.049).

**TABLE 3:** Cardiovascular parameters for SCG and HCG at V1 and V2.

Visit 1	HCG *n* = 20	SCG *n* = 26	Group *p*‐value*	Respiration rate *p*‐value
Cardiovascular Measures				
Respiration rate (breath/min)	17.3 (3.0)	19.5 (4.7)	0.093	‐
Tidal Volume (mL)	450.2 (111.3)	431.8 (142.5)	0.927	‐
Minute Ventilation (L/min)	8.3 (2.2)	8.3 (2.0)	0.726	‐
HR (bpm)	67.4 (9.6)	64.4 (8.5)	0.554	0.207
R–R interval (ms)	915.7 (132.4)	950.5 (128.3)	0.651	0.175
MAP (mmHg)	83.1 (8.3)	81.4 (9.1)	0.783	0.759
SBP (mmHg)	114.3 (12.2)	114.9 (14.4)	0.638	0.585
DBP (mmHg)	64.2 (6.6)	60.1 (12.3)	0.326	0.900
Calculated Autonomic Measures				
RMSSD (ms)	76.1 (46.3)	90.4 (52.5)	0.480	0.568
HF HRV (n. u.)	0.55 (0.15)	0.53 (0.17)	0.743	0.700
LF SBPV (mmHg^2^)	7.79 (11.30)	7.92 (4.99)	0.867	0.266
BRS (ms/mmHg)	13.7 (9.0)	15.5 (12.8)	0.357	0.299

Visit 2	*n* = 13	*n* = 17	Group *p*‐value*	Respiration Rate *p*‐value
Cardiovascular Measures				
Respiration rate (breath/min)	17.3 (1.9)	17.9 (3.0)	0.544	‐
Tidal Volume (mL)	392.7 (135.9)	402.8 (82.4)	0.812	‐
Minute Ventilation (L/min)	7.4 (1.9)	7.6 (1.3)	0.739	‐
HR (bpm)	67.8 (11.9)	66.3 (11.9)	0.970	0.485
R–R interval (ms)	906.8 (154.8)	950.7 (161.5)	0.755	0.525
MAP (mmHg)	82.2 (6.6)	79.7 (10.2)	0.660	0.890
SBP (mmHg)	114.6 (10.1)	111.9 (14.6)	0.729	0.644
DBP (mmHg)	63.6 (5.6)	60.1 (7.9)	0.362	0.973
Calculated Autonomic Measures				
RMSSD (ms)	76.5 (48.8)	97.0 (49.0)	0.277	0.044
HF HRV (n. u.)	0.54 (0.21)	0.62 (0.16)	0.057	0.226
LF SBPV (mmHg^2^)	5.87 (3.59)	10.16 (4.81)	**0.018**	0.217
BRS (ms/mmHg)	19.6 (14.3)	15.4 (9.6)	0.562	0.862

*Note*: bold values indicate a significant finding at *p* < 0.025 after performing a Bonferroni correction.

Abbreviations: DBP, diastolic blood pressure; HCG, healthy control group; HF HRV, high frequency component of heart rate variability; HR, heart rate; LF SBPV, low frequency component of systolic blood pressure variability; MAP, mean arterial pressure; n.u., normalized unites; RMSSD, root mean square of successive differences in R–R interval; SBP, systolic blood pressure; SCG, sport concussion group.

* *p*‐values are from linear regressions after controlling for respiration rate (except for the groupwise comparison of respiration rate, tidal volume and minute ventilation); values are presented as means (standard deviations).

Table 4 presents the correlation between symptom category and ANS parameters in SCG at V1 (*n* = 25). A negative, moderate (Spearman's rho between 0.4–0.6) (Schober et al., 2018) correlation was observed between HF HRV and affective symptoms. Positive moderate correlations were observed between LF SBPV and total, cognitive and fatigue symptom severity.

**TABLE 4:** Correlation coefficients between concussion symptoms and cardiovascular ANS parameters at V1 in SCG.

	RMSSD		HF HRV		LF SBPV		BRS	
	Coefficient	*p*‐value	Coefficient	*p*‐value	Coefficient	*p*‐value	Coefficient	*p*‐value
Total	−0.093	0.658	−0.193	0.355	**0.494**	**0.012**	0.008	0.969
Physical	−0.104	0.620	−0.275	0.183	0.310	0.132	0.125	0.560
Cognitive	−0.005	0.981	−0.039	0.854	**0.598**	**0.002**	0.002	0.992
Affective	−0.394	0.051	**−0.504**	**0.010**	−0.007	0.974	0.015	0.943
Fatigue	−0.088	0.677	−0.084	0.689	**0.439**	**0.028**	0.085	0.692

*Note*: Bolded values indicate a significant finding.

Abbreviations: ANS, autonomic nervous system; HF HRV, high frequency component of heart rate variability; LF SBPV, low frequency component of systolic blood pressure variability; RMSSD, root mean square of successive differences in R–R interval differences.

Table 5 presents the comparison of SCG participants who experienced PPCS with those who did not at V1. Mean recovery time for participants without PPCS was 16 ± 6 days, while for participants with PPCS, it was 52 ± 19 days, which was significantly different (*p* < 0.001). Respiration rate was not different between concussed participants who would develop PPCS or not (*p* = 0.318).

**TABLE 5:** Comparison of cardiovascular autonomic parameters between those who developed PPCS or not at V1.

	Typical recovery/ non‐PPCS *n* = 17	Delayed recovery/ PPCS *n* = 9	Group *p*‐value*	Respiration rate *p*‐value
RMSSD (ms)	98.0 (55.0)	76.1 (47.3)	0.475	0.363
HF HRV (n.u.)	0.53 (0.19)	0.50 (0.12)	0.356	0.640
LF SBPV (mmHg^2^)	8.15 (5.41)	7.48 (4.35)	0.757	0.342
BRS (ms/mmHg)	17.8 (15.0)	11.2 (5.5)	0.088	**0.022**

*Note*: bolded values indicate a significant finding at *p* < 0.025; values are presented as means (standard deviations).

Abbreviations: BRS, baroreceptor sensitivity; HF HRV, high frequency component of heart rate variability; LF SBPV, low frequency component of systolic blood pressure variability; PPCS, persisting post‐concussion symptoms; RMSSD, root mean square of successive differences in R–R interval.

* *p*‐values are from linear regressions after controlling for respiration rate.

## 4 DISCUSSION

This analysis comparing resting cardiovascular ANS activity with clinical measures of SRC and recovery has inconclusive yet informative results.

### 4.1 Acute timepoint

We found no differences in resting cardiovascular ANS measures within 10 days of injury or between those who would have delayed recovery or not, suggesting that these measures have limited diagnostic or prognostic use. These results consistent with the majority of previous studies on cardiovascular ANS function in adolescents and adults with SRC (Bishop et al., 2017; Ellingson et al., 2022; Gall et al., 2004; Johnson et al., 2018; La Fountaine et al., 2009, 2019), showing no significant changes in resting RMSSD, HF HRV, LF SBPV or BRS in the acute/sub‐acute period after SRC. This suggests that resting cardiovascular ANS activity remains intact immediately after concussive injury. However, Ellingson et al. (2022). found a controversial finding on cardiovascular SNS function following acute SRC, with LF SBPV decreased in adult athletes compared to their pre‐SRC values. This discrepancy might be explained by the fact that SBPV is affected by a variety of extrinsic and behavioral factors (Kallioinen et al., 2017), such as the differing durations of alcohol and caffeine abstinence before data collection, controlling for RR (Pitzalis et al., 1998), or using a within‐subjects design. The study by LA Fountaine et al. (2019). did not control RR during the assessment and used a case–control design, which is similar to ours, and found unchanged LF SBPV during the acute SRC phase when comparing concussed athletes with non‐injured controls. Our statistical analyses controlled for RR and found that RR does not significantly affect the relationship between LF SBPV and concussion, therefore, the discrepant finding does not appear to stem from a respiratory origin. We did observe a slightly higher, albeit nonsignificant (*p* = 0.093) RR in symptomatic adolescents with no differences in minute ventilation or tidal volume at V1. This may be attributable to concussed participants being symptomatic and in pain, which may have increased their level of anxiety and changed their breathing patterns (Liu et al., 2022). This difference normalized after concussed participants recovered and were asymptomatic, which provides some justification to our interpretation.

### 4.2 Post‐recovery timepoint

After clinical recovery, we found that LF SBPV was greater in the SCG when compared to controls as well as to themselves when they were symptomatic (V1), which may indicate persistent changes in cardiovascular SNS activity in adolescents with SRC. Persisting ANS changes after clinical recovery from SRC, as measured by HRV, has been reported in several publications (Kamins et al., 2017). Our study aligns with prior publications since we also observed a trend (*p* = 0.057) towards statistical significance for higher HF HRV after clinical recovery. However, to the best of our knowledge, no published research has specifically used LF SBPV to examine cardiovascular SNS activity following recovery from SRC in athletes, and this was the only autonomic measure that was statistically different after recovery in our study. Prior literature suggests that cardiovascular ANS dysfunction persists after mTBI, as evidenced by slower HR recovery from a 20‐min aerobic exercise bout (Memmini et al., 2021) and lower resting LF SBPV (Hilz et al., 2016) in participants with a history of mTBI compared to those without, which is the opposite of what we observed. The increased LF SBPV observed following recovery in our study was not attributed to BRS disruption, as BRS remained unchanged during the acute concussion and recovery phases between the two groups or within each group. Similarly, Memmini et al. (2021) found that concussion has long‐term effects on cardiovascular ANS function, with athletes who had a history of two or more concussions exhibiting decreased PNS tone during recovery from a 20‐min aerobic exercise at 60–70% of their maximal target HR, even beyond 6 months post‐injury. Richer et al. (2024). also noted that adolescents with SRC, regardless of symptom improvement after clinical recovery, continued to exhibit autonomic dysfunction as assessed by HR and systolic BP during head‐up tilt tests. Although Memmini et al. (2021) did not specifically examine SNS tone, our findings, together with theirs and those of Richer et al. (2024) suggest that SRC may have long‐term cardiovascular ANS effects that could disrupt autonomic function over an extended period.

### 4.3 Association with symptoms

Another significant finding in our sample is that higher LF SBPV during acute injury phase was moderately associated with higher total symptom severity, and with cognitive and fatigue symptoms specifically (see Table 4 for correlation values and statistics). Lower HF HRV, on the other hand, was moderately associated only with more affective symptoms. These results are in line with other studies showing that affective and cognitive disturbances, regardless of concussion history, are associated with lower HF HRV (Chalmers et al., 2014; Kemp et al., 2010) and higher SBPV (Chiu et al., 2021; Epstein et al., 2013). Taken together, our data and these studies suggest that adolescents acutely after SRC that exhibit lower resting PNS activity and higher SNS activity may experience more emotional and cognitive symptoms related to concussion. However, our analysis is complicated by the fact that HF HRV and LF SBPV values during acute injury phase were not significantly different from healthy controls. This lack of distinction may be attributed to the high variability in these measures among concussed adolescents, as noted in previous research. Specifically, studies have demonstrated that symptomatic and asymptomatic concussed athletes exhibited no significant differences in HF HRV during weekly assessments following acute SRC, as well as at 1, 3, and 6 months post‐symptom resolution (Paniccia et al., 2018). Moreover, while baseline HF HRV (pre‐concussion) values were positively correlated with normalized HF HRV across all post‐injury assessments (Paniccia et al., 2018), this finding underscores the importance of individual variability in determining autonomic responses following SRC. Therefore, our data suggest that this variability may mask the effect of concussion on HRV, highlighting the need for further investigation into the role of individual differences. It is important to note that while the current study does not demonstrate significant prognostic or diagnostic potential for HRV or SBPV changes in SRC, it serves as a preliminary step in understanding autonomic dysfunction post‐concussion. We recommend that future research incorporates an increased frequency of the same assessments over time to reduce variability and enhance the reliability of findings in this population.

While our study observed similar patterns, with emotional and cognitive problems correlated with ANS activity during the acute concussion phase, altered LF SBPV was not correlated with symptoms following recovery. Previous research suggests that ANS dysfunction is associated with decreased cognitive performance after recovery, as assessed by Immediate Post‐Concussion Assessment and Cognitive Test (ImPACT), a validated cognitive battery in SRC (Taylor et al., 2018). This underscores that the PCSS may provide only a cursory assessment of SRC (Merritt et al., 2015), highlighting the need for more specific and sensitive cognitive assessment tools in future studies to fully understand the implications of autonomic measures on emotional function.

## 5 LIMITATIONS

Our study sample constitutes a typical adolescent SRC population with relatively equal distribution of males and females that were seen a mean of 7 days since their injury. In line with the SRC literature, about one third of adolescents in this study developed PPCS (Boucher et al., 2023; Le Sage et al., 2022). Our primary limitation is that this is a case–control comparison instead of a within‐subjects, and measures of autonomic function vary widely from person to person. We did not account for individual factors that may affect HRV and SBPV such as race, percentage of body fat, subcutaneous adipose tissue, and visceral adipose tissue to name a few (Fatisson et al., 2016). Future research should consider controlling these variables if feasible. Another limitation is the wide time duration from time since injury. Of the 26 concussed participants included in the analysis, 6 were seen within the first 5 days of injury, while 20 were seen between 6 and 10 days post‐injury. Due to the small sample size in the first subgroup and the unequal sample sizes between the two groups, we are underpowered to make meaningful comparisons in this subgroup analysis. Future studies with more balanced sample sizes may be better equipped to determine if there are differences in the early acute and sub‐acute phases of injury. Our V2 comparison is confounded by the high variability of recovery times (28 ± 21 days). Deconditioning should not have affected our V1 analysis since the effect of deconditioning typically takes up to 2 weeks to occur in athletes (Ellis et al., 2018; Leddy et al., 2016), however, those who were symptomatic for longer probably experienced some level of aerobic deconditioning which may have affected their ANS tone. Finally, we had to exclude data from three SRC participants and three healthy controls from the V1 analysis due to artifacts exceeding 5% in ECG and PPG data. Additionally, our study lost 9 participants with SRC and 7 healthy controls to follow‐up. The primary reason for this loss to follow‐up is that concussed and control participants reported their inability to allocate time for a 3‐h research visit while attending school and engaging in sports full‐time. This loss, constituting nearly one‐third of the participants, restricts our understanding of potential autonomic function alterations post‐recovery that might have been observed in these individuals.

## 6 CONCLUSIONS

This study found no differences in resting autonomic function in adolescent athletes within 10 days of SRC when compared to matched controls, or between injured athletes who went on to have typical or delayed recovery. Some differences were observed after clinical recovery, specifically that LF SBPV increased after recovery. Lastly, while adolescent athletes were symptomatic, reduced PNS tone (HF HRV) was moderately associated with greater affective symptom severity, and that greater SNS tone (LF SBPV) was moderately associated with increased total, cognitive and fatigue symptom severity. These measures were not different from controls who were not experiencing symptoms, which limits the interpretability of this finding.

## AUTHOR CONTRIBUTIONS

The experiment was conducted at the Concussion Management Clinic and Research Center, University at Buffalo. The contributions of the authors are as follows: Wenjie Ji: Data Curation, Formal Analysis, Writing—Original Draft Preparation. Haley M Chizuk and Sue Ann Sisto: Data Interpretation, Writing—Review and Editing. John J Leddy: Conceptualization, Data Interpretation, and Writing—Review and Editing. Mohammad N Haider: Conceptualization, Data Curation, Formal Analysis, Data Interpretation, and Writing—Review and Editing. All authors have approved the final version of the manuscript and agree to be accountable for all aspects of the work. This includes ensuring that questions related to the accuracy or integrity of any part of the work are appropriately investigated and resolved. Furthermore, we confirm that all individuals who qualify for authorship are listed and that all listed authors meet the criteria for authorship as per the guidelines.

## ETHICS STATEMENT

Human subjects research was performed under guidelines of the Declaration of Helsinki. Written informed consent was obtained from all participants and the study was reviewed by the University at Buffalo Instritutional Review Board (IRB# STUDY00000092).

## FUNDING INFORMATION

Research reported in this publication was supported by the National Institute of Neurological Disorders and Stroke of the National Institutes of Health award number 1R01NS094444, the National Center for Advancing Translational Sciences of the National Institutes of Health award number UL1TR001412 to the University at Buffalo. The recipient of these grants is Dr. John J Leddy. The content is solely the responsibility of the authors and does not necessarily represent the official views of the National Institutes of Health.

## CONFLICT OF INTEREST STATEMENT

The authors declare no conflicts of interest.

## Data Availability

The raw data used for statistical analyses will be made available by the authors, without undue reservation, to any qualified researcher upon request. The data are not publicly archived but can be obtained from the corresponding author upon reasonable request.

## References

[phy270114-bib-0001] Billman, G. E. (2013). The LF/HF ratio does not accurately measure cardiac sympatho‐vagal balance. Frontiers in Physiology, 4, 25–26. 10.3389/fphys.2013.00026 23431279 PMC3576706

[phy270114-bib-0002] Bishop, S. , Dech, R. , Baker, T. , Butz, M. , Aravinthan, K. , & Neary, J. P. (2017). Parasympathetic baroreflexes and heart rate variability during acute stage of sport concussion recovery. Brain Injury, 31(2), 247–259. 10.1080/02699052.2016.1226385 28045562

[phy270114-bib-0003] Boucher, V. , Frenette, J. , Neveu, X. , Tardif, P. A. , Mercier, É. , Chauny, J. M. , Berthelot, S. , Archambault, P. , Lee, J. , Perry, J. J. , McRae, A. , Lang, E. , Moore, L. , Cameron, P. , Ouellet, M. C. , de Guise, E. , Swaine, B. , Émond, M. , & le Sage, N. (2023). Lack of association between four biomarkers and persistent post‐concussion symptoms after a mild traumatic brain injury. Journal of Clinical Neuroscience, 118, 34–43. 10.1016/j.jocn.2023.10.007 37857062

[phy270114-bib-0004] Carter, J. R. (2019). Microneurography and sympathetic nerve activity: a decade‐by‐decade journey across 50 years. Journal of Neurophysiology, 121(4), 1183–1194. 10.1152/jn.00570.2018 30673363 PMC6485724

[phy270114-bib-0005] Chalmers, J. A. , Quintana, D. S. , Abbott, M. J. , & Kemp, A. H. (2014). Anxiety disorders are associated with reduced heart rate variability: A meta‐analysis. Frontiers in Psychiatry, 5, 80. 10.3389/fpsyt.2014.00080 25071612 PMC4092363

[phy270114-bib-0006] Chiu, T. J. , Yeh, J. T. , Huang, C. J. , Chiang, C. E. , Sung, S. H. , Chen, C. H. , & Cheng, H. M. (2021). Blood pressure variability and cognitive dysfunction: A systematic review and meta‐analysis of longitudinal cohort studies. Journal of Clinical Hypertension, 23(8), 1463–1482. 10.1111/jch.14310 34153171 PMC8678719

[phy270114-bib-0007] Chong, C. D. , & Schwedt, T. J. (2018). Research imaging of brain structure and function after concussion. Headache, 58(6), 827–835. 10.1111/head.13269 29476532

[phy270114-bib-0008] Coffman, C. A. , Kay, J. J. M. , Saba, K. M. , Harrison, A. T. , Holloway, J. P. , LaFountaine, M. F. , & Moore, R. D. (2021). Predictive value of subacute heart rate variability for determining outcome following adolescent concussion. Journal of Clinical Medicine, 10(1), 161. 10.3390/jcm10010161 33466532 PMC7796512

[phy270114-bib-0009] Cowan, M. J. (1995). Measurement of heart rate variability. Western Journal of Nursing Research, 17(1), 32–48. 10.1177/019394599501700104 7863645

[phy270114-bib-0010] Darling, S. R. , Leddy, J. J. , Baker, J. G. , Williams, A. J. , Surace, A. , Miecznikowski, J. C. , & Willer, B. (2014). Evaluation of the Zurich guidelines and exercise testing for return to play in adolescents following concussion. Clinical Journal of Sport Medicine, 24(2), 128–133. 10.1097/JSM.0000000000000026 24184849

[phy270114-bib-0011] Diedrich, A. , Jordan, J. , Tank, J. , Shannon, J. R. , Robertson, R. , Luft, F. C. , Robertson, D. , & Biaggioni, I. (2003). The sympathetic nervous system in hypertension: Assessment by blood pressure variability and ganglionic blockade. Journal of Hypertension, 21(9), 1677–1686. 10.1097/00004872-200309000-00017 12923400

[phy270114-bib-0012] Electrophysiology TFotESoCtNASoP . (1996). Heart rate variability: Standards of measurement, physiological interpretation, and clinical use. Circulation, 93(5), 1043–1065. 10.1161/01.CIR.93.5.1043 8598068

[phy270114-bib-0013] Ellingson, C. J. , Singh, J. , Ellingson, C. A. , Sirant, L. W. , Krätzig, G. P. , Dorsch, K. D. , Piskorski, J. , & Neary, J. P. (2022). Alterations in baroreflex sensitivity and blood pressure variability following sport‐related concussion. Lifestyles, 12(9), 1400. 10.3390/life12091400 PMC9500648 36143435

[phy270114-bib-0014] Ellis, M. J. , Leddy, J. , Cordingley, D. , & Willer, B. (2018). A physiological approach to assessment and rehabilitation of acute concussion in collegiate and professional athletes. Frontiers in Neurology, 9, 1115. 10.3389/fneur.2018.01115 30619068 PMC6306465

[phy270114-bib-0015] Epstein, N. U. , Lane, K. A. , Farlow, M. R. , Risacher, S. L. , Saykin, A. J. , & Gao, S. (2013). Cognitive dysfunction and greater visit‐to‐visit systolic blood pressure variability. Journal of the American Geriatrics Society, 61(12), 2168–2173. 10.1111/jgs.12542 24479146 PMC3923497

[phy270114-bib-0016] Fatisson, J. , Oswald, V. , & Lalonde, F. (2016). Influence diagram of physiological and environmental factors affecting heart rate variability: An extended literature overview. Heart International, 11(1), e32–e40. 10.5301/heartint.5000232 27924215 PMC5056628

[phy270114-bib-0017] Gall, B. , Parkhouse, W. , & Goodman, D. (2004). Heart rate variability of recently concussed athletes at rest and exercise. Medicine and Science in Sports and Exercise, 36(8), 1269–1274. 10.1249/01.Mss.0000135787.73757.4d 15292731

[phy270114-bib-0018] Gulli, G. , Cooper, V. L. , Claydon, V. , & Hainsworth, R. (2003). Cross‐spectral analysis of cardiovascular parameters whilst supine may identify subjects with poor orthostatic tolerance. Clinical Science, 105(1), 119–126. 10.1042/CS20020322 12670299

[phy270114-bib-0019] Haider, M. N. , Leddy, J. J. , Pavlesen, S. , Kluczynski, M. , Baker, J. G. , Miecznikowski, J. C. , & Willer, B. S. (2017). A systematic review of criteria used to define recovery from sport‐related concussion in youth athletes. British Journal of Sports Medicine, 52, 1179–1190. 28735282 10.1136/bjsports-2016-096551 PMC5818323

[phy270114-bib-0020] Heyer, G. L. , Fischer, A. , Wilson, J. , MacDonald, J. , Cribbs, S. , Ravindran, R. , Pommering, T. L. , & Cuff, S. (2016). Orthostatic intolerance and autonomic dysfunction in youth with persistent Postconcussion symptoms: A head‐upright tilt Table study. Clinical Journal of Sport Medicine, 26(1), 40–45. 10.1097/JSM.0000000000000183 25706664

[phy270114-bib-0021] Hilz, M. J. , & Dutsch, M. (2006). Quantitative studies of autonomic function. Muscle & Nerve, 33(1), 6–20. 10.1002/mus.20365 15965941

[phy270114-bib-0022] Hilz, M. J. , Liu, M. , Koehn, J. , Wang, R. , Ammon, F. , Flanagan, S. R. , & Hösl, K. M. (2016). Valsalva maneuver unveils central baroreflex dysfunction with altered blood pressure control in persons with a history of mild traumatic brain injury. BMC Neurology, 16, 61. 10.1186/s12883-016-0584-5 27146718 PMC4857428

[phy270114-bib-0023] Hinds, A. , Leddy, J. , Freitas, M. , Czuczman, N. , & Willer, B. (2016). The effect of exertion on heart rate and rating of perceived exertion in acutely concussed individuals. Journal of neurology & neurophysiology, 7(4), 388. 10.4172/2155-9562.1000388 27812398 PMC5089811

[phy270114-bib-0024] Houle, M. S. , & Billman, G. E. (1999). Low‐frequency component of the heart rate variability spectrum: A poor marker of sympathetic activity. The American Journal of Physiology, 276(1), H215–H223. 10.1152/ajpheart.1999.276.1.H215 9887035

[phy270114-bib-0025] Johnson, B. D. , O'Leary, M. C. , McBryde, M. , Sackett, J. R. , Schlader, Z. J. , & Leddy, J. J. (2018). Face cooling exposes cardiac parasympathetic and sympathetic dysfunction in recently concussed college athletes. Physiological Reports, 6(9), e13694. 29741235 10.14814/phy2.13694 PMC5941219

[phy270114-bib-0026] Johnson, B. D. , Sackett, J. R. , Schlader, Z. J. , & Leddy, J. J. (2020). Attenuated cardiovascular responses to the cold pressor test in concussed collegiate athletes. Journal of Athletic Training, 55, 124–131. 31909640 10.4085/1062-6050-573-18 PMC7017893

[phy270114-bib-0027] Joyner, M. J. (2016). Preclinical and clinical evaluation of autonomic function in humans. The Journal of Physiology, 594(14), 4009–4013. 10.1113/JP271875 27098282 PMC4945718

[phy270114-bib-0028] Kallioinen, N. , Hill, A. , Horswill, M. S. , Ward, H. E. , & Watson, M. O. (2017). Sources of inaccuracy in the measurement of adult patients' resting blood pressure in clinical settings: A systematic review. Journal of Hypertension, 35(3), 421–441. 10.1097/HJH.0000000000001197 27977471 PMC5278896

[phy270114-bib-0029] Kamins, J. , Bigler, E. , Covassin, T. , Henry, L. , Kemp, S. , Leddy, J. J. , Mayer, A. , McCrea, M. , Prins, M. , Schneider, K. J. , Valovich McLeod, T. C. , Zemek, R. , & Giza, C. C. (2017). What is the physiological time to recovery after concussion? Systematic review. British Journal of Sports Medicine, 51, 935–940. 28455363 10.1136/bjsports-2016-097464

[phy270114-bib-0030] Karr, J. E. , Zuccato, B. G. , Ingram, E. O. , McAuley, T. L. , Merker, B. , & Abeare, C. A. (2023). The post‐concussion symptom scale: Normative data for adolescent student‐athletes stratified by gender and preexisting conditions. The American Journal of Sports Medicine, 51(1), 225–236. 10.1177/03635465221131987 36427014

[phy270114-bib-0031] Kemp, A. H. , Quintana, D. S. , Gray, M. A. , Felmingham, K. L. , Brown, K. , & Gatt, J. M. (2010). Impact of depression and antidepressant treatment on heart rate variability: a review and meta‐analysis. Biological Psychiatry, 67(11), 1067–1074. 10.1016/j.biopsych.2009.12.012 20138254

[phy270114-bib-0032] Kumar, A. , Kara, S. , van der Werf, B. , & Fulcher, M. (2023). Can the buffalo concussion treadmill test be used as a prognostic indicator for patients with sport‐related mild traumatic brain injury? Clinical Journal of Sport Medicine, 34(2), 91–96. 10.1097/JSM.0000000000001170 37389465

[phy270114-bib-0033] La Fountaine, M. F. , Heffernan, K. S. , Gossett, J. D. , Bauman, W. A. , & De Meersman, R. E. (2009). Transient suppression of heart rate complexity in concussed athletes. Autonomic Neuroscience, 148(1_2), 101–103. 10.1016/j.autneu.2009.03.001 19303821

[phy270114-bib-0034] LA Fountaine, M. F. , Hohn, A. N. , Testa, A. J. , & Weir, J. P. (2019). Attenuation of spontaneous baroreceptor sensitivity after concussion. Medicine and Science in Sports and Exercise, 51(4), 792–797. 10.1249/MSS.0000000000001833 30407273

[phy270114-bib-0035] La Rovere, M. T. , Pinna, G. D. , & Raczak, G. (2008). Baroreflex sensitivity: Measurement and clinical implications. Annals of Noninvasive Electrocardiology, 13(2), 191–207. 10.1111/j.1542-474X.2008.00219.x 18426445 PMC6931942

[phy270114-bib-0036] Langdon, S. , Königs, M. , Adang, E. , Goedhart, E. , & Oosterlaan, J. (2020). Subtypes of sport‐related concussion: A systematic review and meta‐cluster analysis. Sports Medicine, 50(10), 1829–1842. 32720230 10.1007/s40279-020-01321-9 PMC7497426

[phy270114-bib-0037] Langevin, P. , Fremont, P. , Fait, P. , & Roy, J. S. (2022). Responsiveness of the post‐concussion symptom scale to monitor clinical recovery after concussion or mild traumatic brain injury. Orthopaedic Journal of Sports Medicine, 10(10), 23259671221127049. 10.1177/23259671221127049 36250029 PMC9561659

[phy270114-bib-0038] Le Sage, N. , Chauny, J. M. , Berthelot, S. , Archambault, P. , Neveu, X. , Moore, L. , Boucher, V. , Frenette, J. , De Guise, É. , Ouellet, M. C. , Lee, J. , McRae, A. D. , Lang, E. , Émond, M. , Mercier, É. , Tardif, P. A. , Swaine, B. , Cameron, P. , & Perry, J. J. (2022). Post‐concussion symptoms rule: Derivation and validation of a clinical decision rule for early prediction of persistent symptoms after a mild traumatic brain injury. Journal of Neurotrauma, 39(19–20), 1349–1362. 10.1089/neu.2022.0026 35765917 PMC9529302

[phy270114-bib-0039] Leddy, J. , Hinds, A. , Sirica, D. , & Willer, B. (2016). The role of controlled exercise in concussion management. PM & R: The Journal of Injury, Function, and Rehabilitation, 8(3 Suppl), S91–S100. 10.1016/j.pmrj.2015.10.017 26972272

[phy270114-bib-0040] Liu, S. , Ye, M. , Pao, G. M. , Song, S. M. , Jhang, J. , Jiang, H. , Kim, J. H. , Kang, S. J. , Kim, D. I. , & Han, S. (2022). Divergent brainstem opioidergic pathways that coordinate breathing with pain and emotions. Neuron, 110(5), 857–873. 34921781 10.1016/j.neuron.2021.11.029 PMC8897232

[phy270114-bib-0041] Lucci, V. M. , Inskip, J. A. , McGrath, M. S. , Ruiz, I. , Lee, R. , Kwon, B. K. , & Claydon, V. E. (2021). Longitudinal assessment of autonomic function during the acute phase of spinal cord injury: Use of low‐frequency blood pressure variability as a quantitative measure of autonomic function. Journal of Neurotrauma, 38(3), 309–321. 10.1089/neu.2020.7286 32940126

[phy270114-bib-0042] Malik, M. , & Camm, A. J. (1993). Components of heart rate variability—what they really mean and what we really measure. The American Journal of Cardiology, 72(11), 821–822. 10.1016/0002-9149(93)91070-x 8093124

[phy270114-bib-0043] McCrea, M. , Meier, T. , Huber, D. , Ptito, A. , Bigler, E. , Debert, C. T. , Manley, G. , Menon, D. , Chen, J. K. , Wall, R. , Schneider, K. J. , & McAllister, T. (2017). Role of advanced neuroimaging, fluid biomarkers and genetic testing in the assessment of sport‐related concussion: A systematic review. British Journal of Sports Medicine, 51(12), 919–929. 10.1136/bjsports-2016-097447 28455364

[phy270114-bib-0044] McCrory, P. , Meeuwisse, W. , Dvořák, J. , Aubry, M. , Bailes, J. , Broglio, S. , Cantu, R. C. , Cassidy, D. , Echemendia, R. J. , Castellani, R. J. , Davis, G. A. , Ellenbogen, R. , Emery, C. , Engebretsen, L. , Feddermann‐Demont, N. , Giza, C. C. , Guskiewicz, K. M. , Herring, S. , Iverson, G. L. , … Vos, P. E. (2017). Consensus statement on concussion in sport‐the 5(th) international conference on concussion in sport held in Berlin, October 2016. British Journal of Sports Medicine, 51(11), 838–847. 10.1136/bjsports-2017-097699 28446457

[phy270114-bib-0045] Memmini, A. K. , La Fountaine, M. F. , Broglio, S. P. , & Moore, R. D. (2021). Long‐Term Influence of Concussion on Cardio‐Autonomic Function in Adolescent Hockey Players. Journal of Athletic Training, 56(2), 141–147. 10.4085/1062-6050-0578.19 33400783 PMC7901578

[phy270114-bib-0046] Mercier, L. J. , Batycky, J. , Campbell, C. , Schneider, K. , Smirl, J. , & Debert, C. T. (2022). Autonomic dysfunction in adults following mild traumatic brain injury: A systematic review. NeuroRehabilitation, 50(1), 3–32. 10.3233/NRE-210243 35068421

[phy270114-bib-0047] Merritt, V. C. , Meyer, J. E. , & Arnett, P. A. (2015). A novel approach to classifying postconcussion symptoms: The application of a new framework to the post‐concussion symptom scale. Journal of Clinical and Experimental Neuropsychology, 37(7), 764–775. 26241079 10.1080/13803395.2015.1060950

[phy270114-bib-0048] Metelka, R. (2014). Heart rate variability—current diagnosis of the cardiac autonomic neuropathy. A review. Biomedical Papers of the Medical Faculty of the University Palacky, Olomouc, Czech Republic, 158(3), 327–338. 10.5507/bp.2014.025 25004914

[phy270114-bib-0049] Ondrusova, K. , Svacinova, J. , Javorka, M. , Novak, J. , Novakova, M. , & Novakova, Z. (2017). Impaired baroreflex function during orthostatic challenge in patients after spinal cord injury. Journal of Neurotrauma, 34(24), 3381–3387. 10.1089/neu.2017.4989 28605971

[phy270114-bib-0050] Paniccia, M. , Verweel, L. , Thomas, S. G. , Taha, T. , Keightley, M. , Wilson, K. E. , & Reed, N. (2018). Heart rate variability following youth concussion: How do autonomic regulation and concussion symptoms differ over time postinjury. BMJ Open Sport & Exercise Medicine, 4(1), 1–9. 10.1136/bmjsem-2018-000355 PMC6173244 30305921

[phy270114-bib-0051] Parati, G. , Di Rienzo, M. , & Mancia, G. (2000). How to measure baroreflex sensitivity: From the cardiovascular laboratory to daily life. Journal of Hypertension, 18(1), 7–19. 10678538

[phy270114-bib-0052] Parati, G. , Stergiou, G. S. , Dolan, E. , & Bilo, G. (2018). Blood pressure variability: Clinical relevance and application. Journal of Clinical Hypertension, 20(7), 1133–1137. 10.1111/jch.13304 30003704 PMC8030809

[phy270114-bib-0053] Patricios, J. S. , Schneider, K. J. , Dvorak, J. , Ahmed, O. H. , Blauwet, C. , Cantu, R. C. , Davis, G. A. , Echemendia, R. J. , Makdissi, M. , McNamee, M. , Broglio, S. , Emery, C. A. , Feddermann‐Demont, N. , Fuller, G. W. , Giza, C. C. , Guskiewicz, K. M. , Hainline, B. , Iverson, G. L. , Kutcher, J. S. , … Meeuwisse, W. (2023). Consensus statement on concussion in sport: The 6th international conference on concussion in sport–Amsterdam, October 2022. British Journal of Sports Medicine, 57(11), 695–711. 37316210 10.1136/bjsports-2023-106898

[phy270114-bib-0054] Pitzalis, M. V. , Mastropasqua, F. , Massari, F. , Passantino, A. , Colombo, R. , Mannarini, A. , Forleo, C. , & Rizzon, P. (1998). Effect of respiratory rate on the relationships between RR interval and systolic blood pressure fluctuations: A frequency‐dependent phenomenon. Cardiovascular Research, 38(2), 332–339. 10.1016/s0008-6363(98)00029-7 9709393

[phy270114-bib-0055] Polak, P. , Leddy, J. J. , Dwyer, M. G. , Willer, B. , & Zivadinov, R. (2015). Diffusion tensor imaging alterations in patients with postconcussion syndrome undergoing exercise treatment: A pilot longitudinal study. The Journal of Head Trauma Rehabilitation, 30(2), E32–E42. 10.1097/HTR.0000000000000037 24721808

[phy270114-bib-0056] Purkayastha, S. , Williams, B. , Murphy, M. , Lyng, S. , Sabo, T. , & Bell, K. R. (2019). Reduced heart rate variability and lower cerebral blood flow associated with poor cognition during recovery following concussion. Autonomic Neuroscience, 220, 102548. 10.1016/j.autneu.2019.04.004 31331690

[phy270114-bib-0057] Richer, L. , Craig, W. , Linsdell, M. , Tang, K. , & Zemek, R. (2024). Autonomic Cardioregulatory function does not correlate with symptom improvement after concussion in children and adolescents. Journal of Neurotrauma, 41(1–2), 161–170. 10.1089/neu.2023.0113 37310894

[phy270114-bib-0058] Schober, P. , Boer, C. , & Schwarte, L. A. (2018). Correlation coefficients: Appropriate use and interpretation. Anesthesia and Analgesia, 126(5), 1763–1768. 10.1213/ANE.0000000000002864 29481436

[phy270114-bib-0059] Stauss, H. M. (2003). Heart rate variability. American Journal of Physiology, 285(5), 927–931. 10.1152/ajpregu.00452.2003 14557228

[phy270114-bib-0060] Strachan, N. C. (2013). Baroreceptor Sensitivity And Heart Rate Variability In Sport Related Concussions. The University Of British Columbia.

[phy270114-bib-0061] Tarvainen, M. P. , Ranta‐Aho, P. O. , & Karjalainen, P. A. (2002). An advanced detrending method with application to HRV analysis. IEEE Trans Biomed Eng, 49(2), 172–175. 10.1109/10.979357 12066885

[phy270114-bib-0062] Taylor, K. M. , Kioumourtzoglou, M. A. , Clover, J. , Coull, B. A. , Dennerlein, J. T. , Bellinger, D. C. , & Weisskopf, M. G. (2018). Concussion history and cognitive function in a large cohort of adolescent athletes. The American Journal of Sports Medicine, 46(13), 3262–3270. 10.1177/0363546518798801 30230912 PMC6940017

[phy270114-bib-0063] Thayer, J. F. , & Lane, R. D. (2009). Claude Bernard and the heart‐brain connection: Further elaboration of a model of neurovisceral integration. Neuroscience and Biobehavioral Reviews, 33(2), 81–88. 10.1016/j.neubiorev.2008.08.004 18771686

